# Contact Force and Scanning Velocity during Active Roughness Perception

**DOI:** 10.1371/journal.pone.0093363

**Published:** 2014-03-27

**Authors:** Yoshihiro Tanaka, Wouter M. Bergmann Tiest, Astrid M. L. Kappers, Akihito Sano

**Affiliations:** 1 Department of Engineering Physics, Electronics and Mechanics, Graduate School of Engineering, Nagoya Institute of Technology, Nagoya, Japan; 2 Helmholtz Institute, Utrecht University, Utrecht, The Netherlands; University of Ottawa, Canada

## Abstract

Haptic perception is bidirectionally related to exploratory movements, which means that exploration influences perception, but perception also influences exploration. We can optimize or change exploratory movements according to the perception and/or the task, consciously or unconsciously. This paper presents a psychophysical experiment on active roughness perception to investigate movement changes as the haptic task changes. Exerted normal force and scanning velocity are measured in different perceptual tasks (discrimination or identification) using rough and smooth stimuli. The results show that humans use a greater variation in contact force for the smooth stimuli than for the rough stimuli. Moreover, they use higher scanning velocities and shorter break times between stimuli in the discrimination task than in the identification task. Thus, in roughness perception humans spontaneously use different strategies that seem effective for the perceptual task and the stimuli. A control task, in which the participants just explore the stimuli without any perceptual objective, shows that humans use a smaller contact force and a lower scanning velocity for the rough stimuli than for the smooth stimuli. Possibly, these strategies are related to aversiveness while exploring stimuli.

## Introduction

Haptic perception is bidirectionally related to exploratory movements. The exploratory movements generate the stimulation from which perception is derived and perception influences the movements. We can optimize or change the exploratory movements according to the perception and/or the task, consciously or unconsciously. The bidirectional characteristics contribute not only to tactile exploration but also to object handling. For example, when humans lift and manipulate objects, they adjust their grip forces to prevent the object from slipping, while avoiding the use of excessive grip forces [1]. Slipping of the fingers over the object is an essential component of tactile exploration. The behavioral objectives are quite different between object handling and tactile exploration and consequently the meaning of feedback of tactile signals is also different. In this paper, the bidirectionality of tactile exploration, especially roughness perception, will be discussed.

Lederman and Klatzky [2] showed that humans select the type of exploratory movement according to the type of tactual information which they want to obtain. This finding is a qualitative aspect of bidirectionality in tactile exploration. Recently, several psychophysical studies have investigated exploratory movements quantitatively for haptic perception. Gamzu and Ahissar [3] reported that some participants change the exploratory speed according to grating frequency when scanning a textured surface with their fingers. Smith, Gosselin, and Houde [4] showed that participants use smaller contact forces when searching for a raised square on a plate as compared to searching for a recessed square. Kaim and Drewing [5] reported adaptation of exerted finger force to the softness of stimuli. Drewing, Lezkan, and Ludwig [6] focused on the number of strokes. They showed that the more often and longer participants obtained redundant information, the better they were able to discriminate between two gratings and that the participants adapt their exploratory behavior towards optimal when they have experience with the perceptual task. Some brain studies reported that the activated area in the human brain is different for different types of target tactual information such as shape and roughness [7], or different objectives such as whether or not roughness estimation is required during tactile exploration [8]. In the present study, we would like to investigate whether such modifications of strategy also play a role in roughness perception, and how they depend on the perceptual task.

Studying the bidirectionality of human haptic perception is important for understanding tactual perception and might give useful knowledge for tactile device development, products design, skill training, etc. For example, emulating human exploratory movements with actuators carrying tactile sensors could enhance the dynamic range, resolution, or environmental adaptation of the sensors when measuring physical properties in the same way as the human sensory system. Performance of tactile displays could be optimized by adapting them better to the human user on the basis of a bidirectional relationship between the exploratory movements and desired tactile sensations. Based on the finding of different types of exploratory movements by Lederman and Klatzky [2], Sinapov, Sukhoy, Sahai, and Stoytchev [9] developed a tactile sensing system utilizing a robot arm and showed that by applying several different exploratory behaviors on a test surface, the robot could recognize surfaces better than with any single behavior alone. Tanaka, Horita, Sano, and Fujimoto [10] proposed a tactile sensing system consisting of microphones mounted on the human finger. This sensor includes bidirectionality on haptic perception since humans can explore while directly contacting the object with the bare finger pad. Humans can evaluate roughness for surfaces with various shapes by adapting the exploratory movements of their fingers to the shape. Experimental results showed that the sensor could evaluate roughness on a curved surface as well as on a flat surface. More recently, Tanaka, Horita, and Sano [11] used a finger-mounted sensor to measure the characteristics of vibrations transmitted by the skin. In order to further improve such sensors, it is important to extend our knowledge on the bidirectional relationship between exploratory movements, the stimulus characteristics, and the type of desired perceptual information.

Roughness or texture perception has been studied in different ways. Perceived roughness has been found to depend on physical roughness [12] and also friction [13], among other things. Psychophysical experiments using a precisely controlled surface like the spacing between and the height of surface elements demonstrated that perceived roughness was strongly related to the spatial deformation of the fingertip's skin by the grooves of the texture [14], [15], [16], [17]. Recent psychophysical and brain studies indicate that roughness perception of fine-textured surfaces, with spatial periods below 200 μm, is derived from temporal stimuli based on the vibration elicited in the skin [18], [19], [20], [21]. In addition, the influence of the exploratory movements on perceived roughness has been investigated. Lederman [22] showed that roughness estimated by passive touch is not different from that by active touch. Subjective roughness estimation has been shown to be affected by the contact force through an experiment with controlled finger force [16]. Psychophysical experiments using stimuli with controlled groove width demonstrated that the scanning velocity of the finger had a negligible effect on perceived roughness [14], [23], whereas Cascio and Sathian [24] demonstrated that roughness magnitude estimates depended on both groove width and scanning velocity through experiments using stimuli with controlled groove width and ridge width. These experiments demonstrated contradictory results on the influence of the velocity. Cascio and Sathian [24] discussed that one reason for this discrepancy is their inclusion of surfaces characterised by differences in ridge width, which elicited the clearest temporal effects, whereas the earlier studies varied groove width. The present study will investigate how the scanning velocity during roughness perception depends on the particular task.

Parameters of the exploratory movements, such as the exerted force, velocity, etc. have often been controlled in many psychophysical experiments on haptic perception. There is no doubt that controlling the behavior is effective for studying the influence and effects of the exploratory parameters on haptic perception. But in realistic situations, tactual exploration is active and haptic perception includes bidirectionality as mentioned before. It is difficult to study this bidirectionality under controlled conditions only. Psychophysical studies sometimes utilized spontaneous touch behavior [15], [25], [26]. Most of these studies have investigated the relationship between the roughness estimate and the deformation or forces on the finger pad derived from the stimuli, or the change in roughness estimate due to different exploratory movements. Smith and Scott [27] reported that participants maintained a relatively constant normal force in the subjective scaling of smooth surface friction. In their experiment, just a single group of smooth surfaces was used and they investigated the exploratory movements. A comparison with exploratory movements used in other ranges of stimulation or other perceptual objectives has not been investigated. This study investigates how humans might change or optimize their exploratory movements for roughness perception as a result of the bidirectionality.

To summarize, the objective of the current study is to investigate the influence of the type of stimulus and perceptual task on exploratory movements during roughness perception. Our hypothesis is that humans change their exploratory movements used in roughness perception according to the type of stimulus or perceptual objective in order to enhance their roughness rating performance. Differences in exploratory movements between perceptual tasks with different types of stimuli or different objectives may be reasonably expected. In particular, we have focused on the influence of the perceptual task (identification and discrimination) and of the roughness of the stimuli on the movements. The identification task and the discrimination task are associated with a classification of perceived intensity and an evaluation of the difference of perceived intensity, respectively. Concerning the intensity of the stimuli, we used a set of rough stimuli and a set of smooth stimuli for each task. The exerted force has been investigated in a previous study [28]. In the present paper, exerted force, the scanning velocity, and the break time between touching stimuli during exploratory behavior are investigated in active roughness perception.

## Methods

### Participants

Eleven healthy adult persons (4 male and 7 female, age range 21–31, mean 25) participated in the experiment. All participants were strongly right-handed according to Coren's test [29]. All participants gave their written informed consent before participating in this study. They were paid for their time. This study was conducted in accordance with principles as stated in the declaration of Helsinki. Participants performed simple psychophysical tasks that did not deviate in stimulus intensity from what is encountered in daily life. As the Medical Ethical Committee of Utrecht University declared that for a similar subject study [PLoS ONE, 7(10): e45298] ethical approval was not necessary, we did not seek formal approval. The Ethical Committee of the Faculty of Human Movement Sciences (ECB) of VU University ascertains that, as far as they can see, the research projects performed in Utrecht seem to be in line with the guidelines of the ECB.

### Stimuli

**Figure 1:**
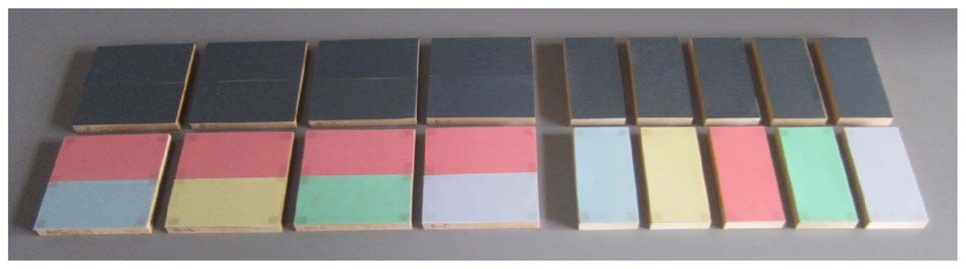
Stimuli. The stimuli were attached to wooden plates (50×100×12 mm). The sandpapers in the upper row belong to the rough group and the lower sandpapers belong to the smooth group. Left: in order for the participant to be able to quickly switch between stimuli, pairs of stimuli were prepared for the discrimination task. Different roughnesses have different colors in the smooth group.

Commercial sandpapers (Lapping Film and waterproof abrasive paper, Fuji Star Coated Abrasives, Inc.) were used as stimuli (see Figure 1). The stimuli were classified into two groups, a smooth group and a rough group, based on their roughness. Each group had 5 stimuli with different grain sizes. The grain size is so different between the smooth group and the rough group that it is easy to discriminate the smooth group from the rough group. Table 1 shows the average grain size of each stimulus. The stimuli were attached to wooden plates (50×100×12 mm).

**Table 1:** Average grain size of the stimuli.

Smooth group		Rough group	
Name	Average grain size (μm)	Name	Average grain size (μm)
S1	1	R1	25
S2	2	R2	30
S3	3	R3	35
S4	5	R4	50
S5	9	R5	55

### Procedure

#### Setup

**Figure 2:**
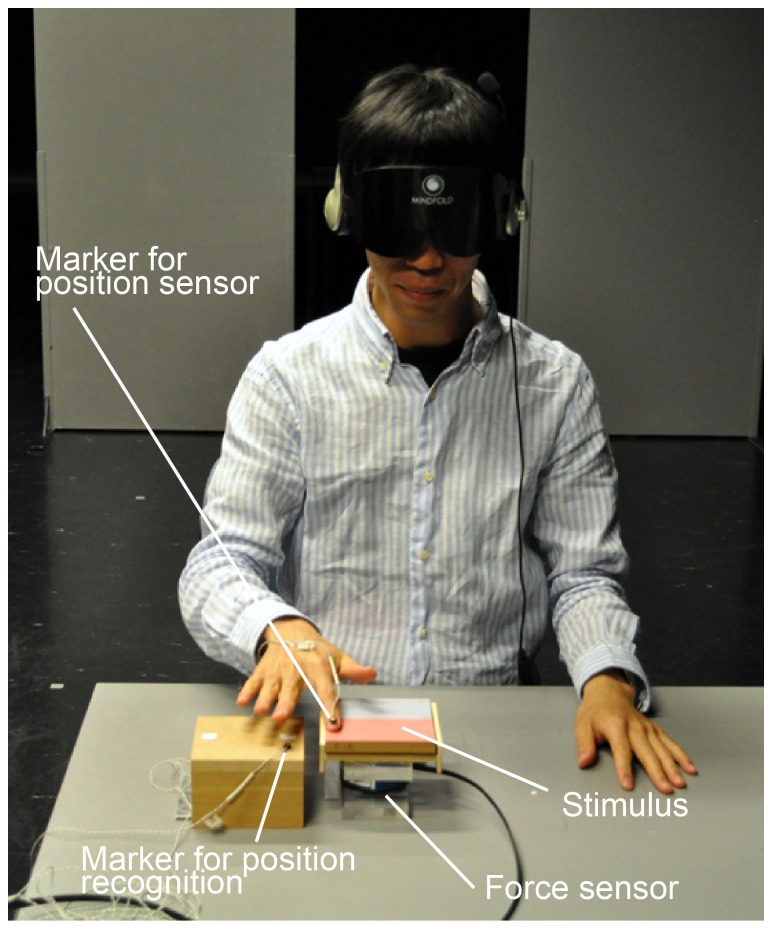
Experimental setup. Exerted force and position of the index finger were measured by the 6-axis force sensor and the 3D optical position sensor, respectively. The person in the photograph is not a subject in the study but is one of the authors demonstrating the procedure.

Figure 2 shows the experimental setup. The individual in this manuscript has given written informed consent (as outlined in PLOS consent form) to publish these case details. The stimuli were placed on a 6-axis force sensor (IFS-67M25A25-I40, Nitta Corporation). In front of the participant, a 3D optical position sensor (Optotrak Certus, NDI) was set up for measurement of the finger position during the experiment. The participants were blindfolded and wore headphones playing white noise so that they could not hear the sound of touching the stimuli. The marker for the 3D optical position sensor was attached to the nail of the index finger of the dominant hand with double-sided tape. Exerted force and position of the finger were measured during the experiment at the sampling frequency of 1 kHz and 500 Hz, respectively. In addition, a board with a marker was placed next to the force sensor. Before each trial, the participants touched that marker with their index finger in order to calibrate the position sensor. During the experiment, the participants were comfortably seated at a table.

The participants had to use the index finger of their dominant hand and rub the stimuli by a back-and-forth motion to the right and to the left. If the participants wanted to touch the stimuli again, they had first to release the contact finger from the stimuli. The participants were not given any instructions on exerted force, velocity, or the distance of the stroke. The plate with the stimulus imposed a limitation on the distance of the stroke. However, it was large enough for the participants to make a comfortable stroke and they touched the stimulus in a natural way.

#### Experimental design

**Figure 3:**
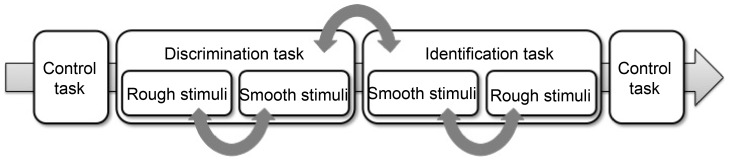
Order of conditions in the experiment. Curved arrows indicate parts to be randomized over participants.

Figure 3 presents an example of the composition of the experiment for one participant. The experiment consisted of 3 kinds of tasks: an identification task, a discrimination task, and a control task. The identification task and the discrimination task had two conditions each: one using the smooth stimuli and one using the rough stimuli. The order of the two conditions in each task was randomized between participants and the order of the identification task and the discrimination task was also randomized between participants. The control task was carried out at the beginning and at the end of the experiment. On average, 1.5 hours per participant were needed to perform the complete experiment (control task: about 3 min/condition, discrimination task: about 20 min/condition, identification task: about 15 min/condition). The participants were allowed to have a break of a few minutes between conditions. In the following section, details of each task are presented.

#### Control task

The objective of a perceptual task might affect the exploratory movements. For comparison with the experimental results in the perceptual tasks, a control task without the objective of rating or discriminating the roughness was conducted.

In this task, the participants did not have to give any response. The participants were instructed to touch the stimuli and just explore them. The participants felt roughness but they were not required to give any judgment of the roughness. They were instructed to make two back-and-forth motions (i.e. four strokes) on each trial. No feedback was given. The stimuli were presented alternately from the smooth group and the rough group. Each stimulus was presented once in a random order, different for each participant, for a total of 10 trials.

#### Discrimination task

Sandpaper S3 of the smooth group and sandpaper R3 of the rough group were used as reference stimuli and the 4 remaining stimuli of a group were used as test stimuli. The roughnesses of the test stimuli were both below and above the reference stimulus.

The participants were presented with pairs of stimuli. A pair always contained a reference stimulus and one of the test stimuli. Each trial, the participants had to judge which of the two stimuli was rougher. The participants were allowed to touch the stimuli as often as they liked, but they were not allowed to touch the same stimulus continuously. After they rubbed one stimulus by a single back-and-forth motion (i.e. two strokes), they had to switch to the other stimulus. No feedback was given.

The positions of the reference stimulus and each test stimulus were randomized (near or far with respect to the participants; see Figure 2). The 4 pairs of stimuli of each condition (smooth group and rough group) were presented 10 times in a random order, different for each participant. Each condition had a total of 40 trials.

#### Identification task

At the beginning of each condition, the roughest stimulus and the smoothest stimulus of the smooth group or the rough group were presented to the participants. The participants touched the stimulus and were instructed to remember that the number of the roughest stimulus and the smoothest stimulus is 5 and 1, respectively. Then, each stimulus was presented and the participants had to rate the presented stimulus on a scale of 1 to 5. Immediately after an answer of the participants, the correct answer was given. The feedback was included in the identification task in order to reinforce the participant's knowledge of the range of stimuli corresponding to their numerical judgment. The participants were allowed to touch the stimuli as often as they liked.

The 5 stimuli of each condition (smooth group and rough group) were presented 5 times in a random order, different for each participant. Each condition had a total of 25 trials.

### Data Processing

**Figure 4:**
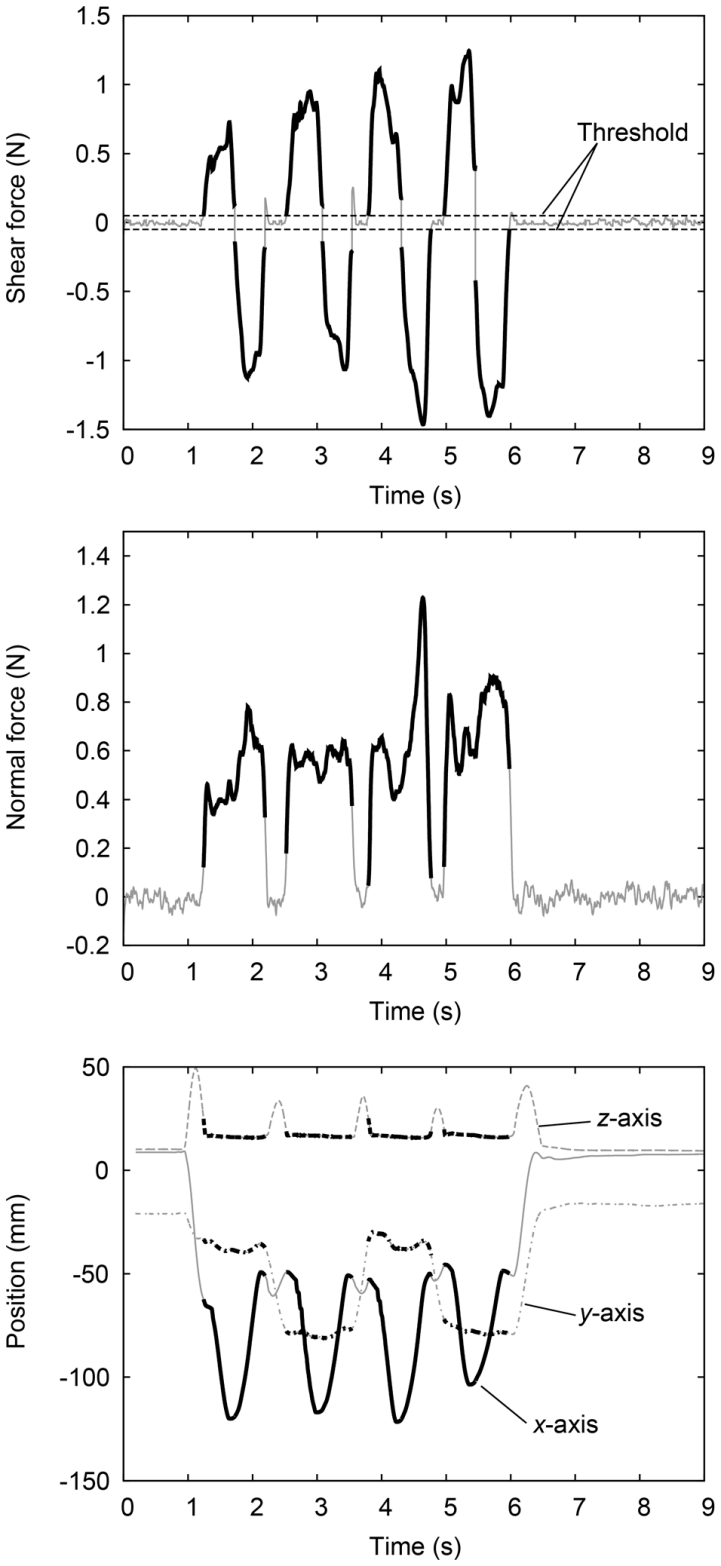
An example of the measurements of exploratory movements. Shear force, normal force, and finger position were measured. An example of data collected during a discrimination trial is shown. The bold parts in each panel indicate the parts extracted for further analysis.

Figure 4 shows a typical example of the data collected in one trial for one participant of the normal force, the shear force, and the finger position. Here, the left-right direction and the anteroposterior direction for the participants were defined as *x*-axis and *y*-axis, respectively. The vertical direction was defined as *z*-axis. Low pass filtering with a cut-off frequency of 10 Hz was applied to the raw force data of each axis for smoothing. Next, the shear force was calculated as a resultant of the *x*-axis and *y*-axis forces and the sign of the shear force was the same as the sign of the *x*-axis force. The normal force was the *z*-axis force. It was difficult to determine the period of scanning a stimulus using the normal force since participants sometimes kept touching the stimulus after scanning. Therefore, the measured shear force was used to select sections of data corresponding to single strokes. A single stroke is defined as a one-directional movement of the finger in contact with the stimulus. The threshold used as a selection criterion was empirically determined to be ±0.05 N of shear force (just above the amplitude of the sensor noise).

**Figure 5:**
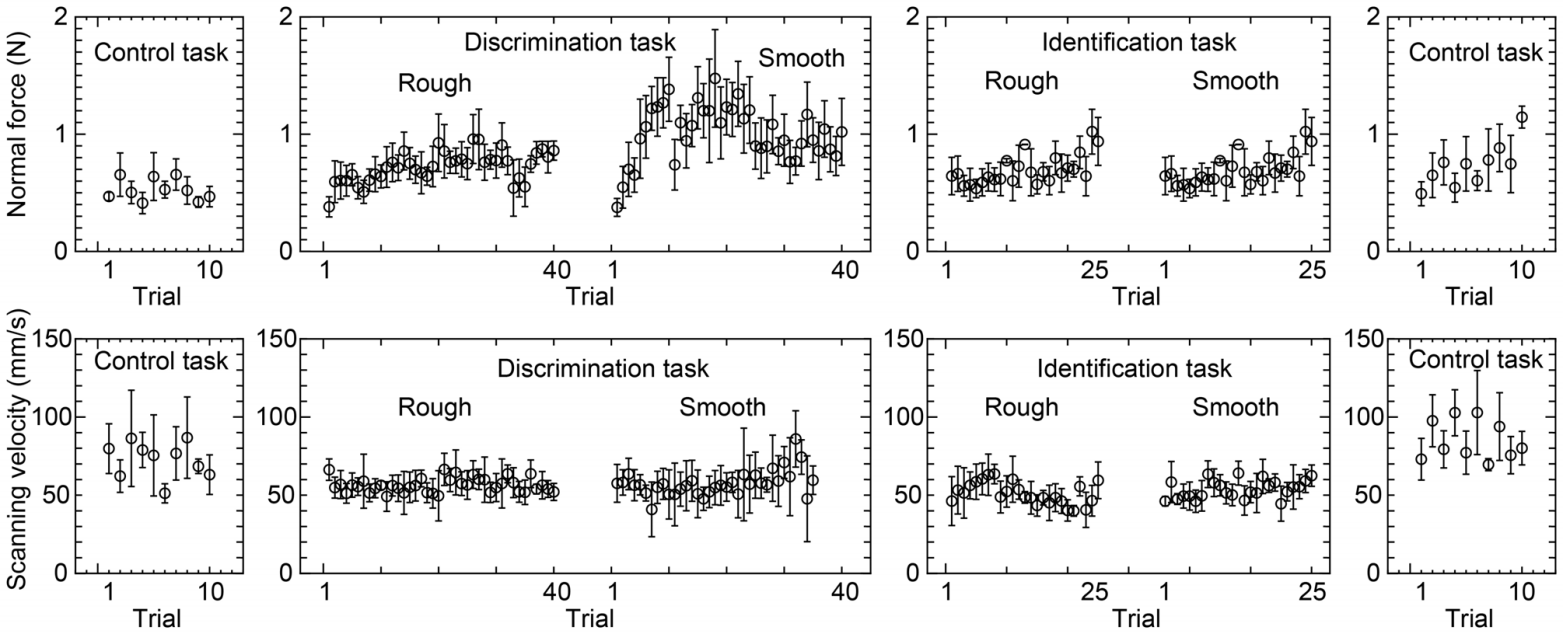
An example of data collected in the experiment for one participant. Each plot shows the average and the standard deviation of the normal force or scanning velocity for each trial.

For each extracted single stroke, the average normal force was calculated. The scanning velocity at each time was calculated using the horizontally moved distance during one sampling (2 ms) calculated from the finger position data. For each extracted single stroke, the average scanning velocity was calculated. Next, the averages of the normal force and scanning velocity for each trial and their standard deviations were calculated using the averages calculated from each extracted profile. An example of the result of one participant is shown in Figure 5. Each plot shows the average and the standard deviation of the normal force or scanning velocity for all trials.

For each participant, 2 parameters relating to the normal force and the scanning velocity were extracted: the average in each condition and the coefficient of variation in each condition. The average in each condition and the coefficient of variation in each condition were calculated using the averages of the force or the velocity in each trial.

In addition to the force and velocity, the break time was measured using the time between the extracted profiles. In the control and identification tasks, the time from the end of scanning a stimulus to the beginning of scanning the stimulus again was calculated. In the discrimination task, the time from the end of scanning the 1^st^ stimulus to the beginning of scanning the 2^nd^ stimulus was used. Next, the average of the break time for each condition was calculated using the average for each trial.

Differences among each condition in each parameter were investigated. For each obtained parameter in all participants, a two-way repeated measures ANOVA with the perceptual task (identification and discrimination) and the stimuli (rough and smooth) as factors, and an ANOVA with the control task (1^st^ and 2^nd^ control condition) and the stimuli as factors were conducted. In this paper, the significance level is set to *α* = 0.05. Before the ANOVAs, a Shapiro-Wilk test and Levene's test were conducted to confirm the assumption of a normal distribution of all dependent parameters and homogeneity of variance across cells. If either the Shapiro-Wilk test or Levene's test was violated, we used the non-parametric Friedman test for comparisons between the 4 conditions in the perceptual task: discrimination-rough (DR), discrimination-smooth (DS), identification-rough (IR), and identification-smooth (IS) or the 4 conditions in the control task: 1^st^ control-rough (C1R), 1^st^ control-smooth (C1S), 2^nd^ control-rough (C2R), and 2^nd^ control-smooth (C2S). In case this test yielded a significant result, 4 Wilcoxon signed rank tests (DR-DS, IR-IS, DR-IR and DS-IS, or C1R-C1S, C2R-C2S, C1R-C2R, and C1S-C2S) were performed in which we corrected for multiple comparisons with a Bonferroni adjustment.

Furthermore, for each trial, the average of the shear force was calculated for each extracted single stroke. Next, the average of the shear force for each trial was calculated using the averages calculated from each single stroke. A friction coefficient for each trial was calculated by dividing the average of the shear force for each trial by the average of the normal force for each trial. In addition, for the perceptual tasks, the number of strokes and the percentage of correct answers were computed. The average number of strokes for each condition and each participant was calculated from all trials in each condition. The percentage of correct answers for each condition and each participant was calculated separately for discrimination and identification tasks with the smooth and the rough stimuli.

## Results

### Normal Force, Scanning Velocity and Break Time

**Figure 6:**
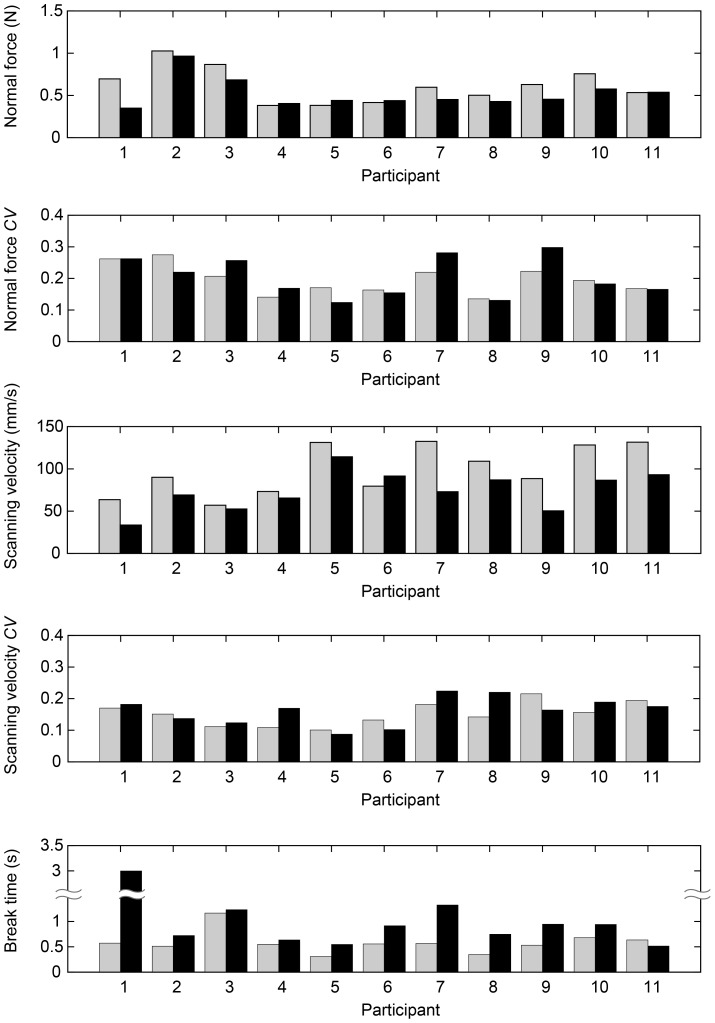
Averages of each parameter for data collected in perceptual tasks for each participant. Gray bars indicate averages in the discrimination task and black bars indicate averages in the identification task.

The average of each parameter relating to the normal force, the scanning velocity and the break time for each perceptual task was calculated for each participant using the averages obtained on both the smooth and the rough stimuli. The results are shown in Figure 6. Gray bars show the results of the discrimination task, and black bars the results of the identification task. In the following subsections, the various parameters will be analyzed statistically for the influence of task and stimulus.

#### Normal force

**Figure 7:**
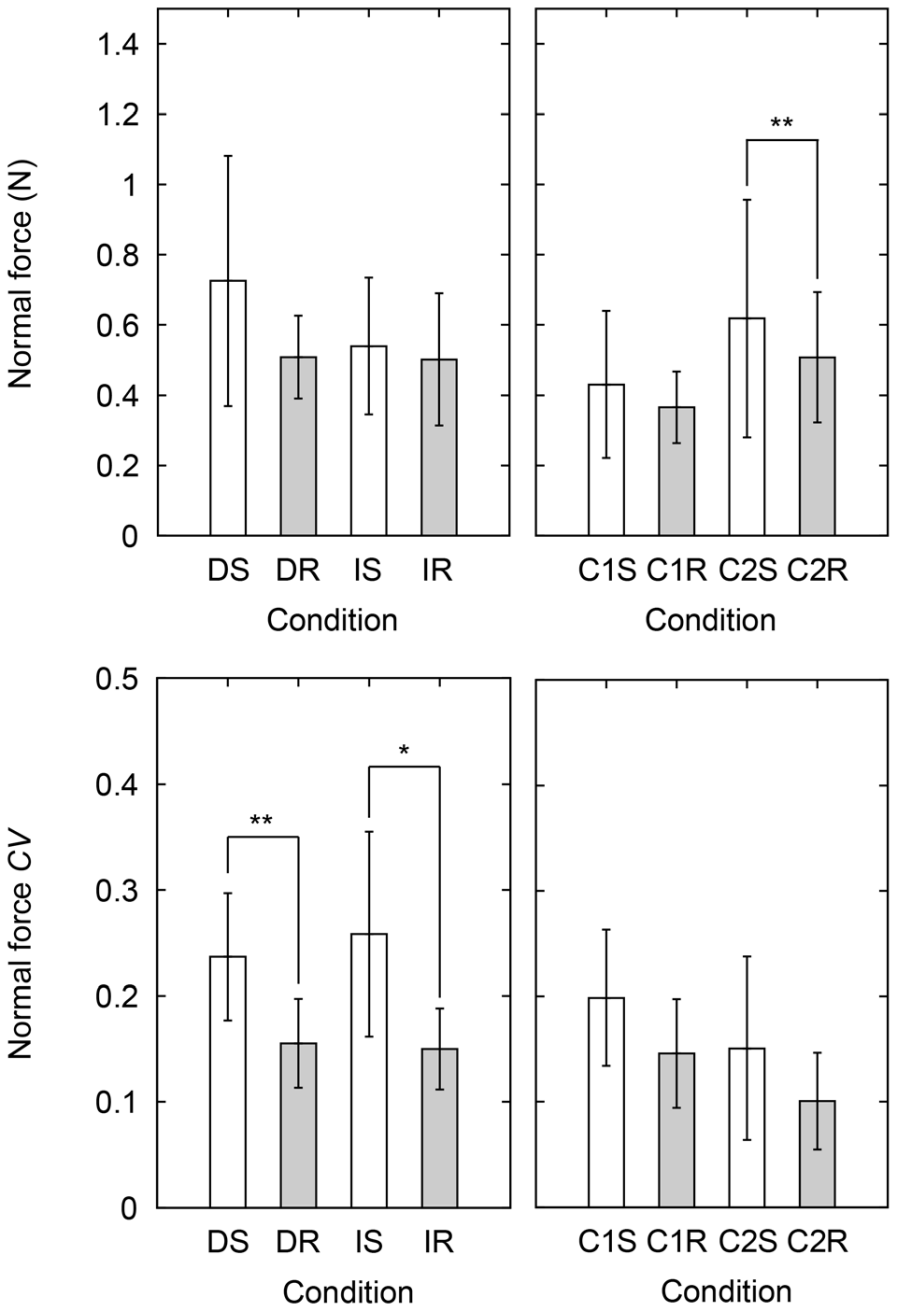
Averages and coefficients of variation of normal force in the various conditions. Left panels: DS, DR, IS, and IR and right panels: C1S, C1R, C2S, and C2R indicate each condition. D, I, C1, and C2 mean discrimination task, identification task, 1^st^ control condition, and 2^nd^ control condition, and S and R mean smooth group and rough group. Error bars indicate the standard deviation. *indicates *p*<0.05 and **p<0.01 with Wilcoxon signed rank tests.

Figure 7 shows the obtained results on the normal force. All of the parameters involving the normal force, i.e. the average normal force and the coefficient of variance in the perceptual task and the control task, violated the Shapiro-Wilk tests. The non-parametric Friedman tests on the average force showed significant differences in the perceptual task (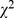 (3) = 8.3, *p* = 0.039) and in the control task (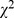
(3) = 15, *p* = 0.0016). The 4 Wilcoxon signed rank tests on the average force showed no significant influence in the perceptual conditions and a significant influence of stimulus type in the 2^nd^ control condition (*W*(11) = 0, *p* = 0.0039). This result shows that the exerted force in the 2^nd^ control condition is larger for the smooth stimuli than for the rough stimuli. The non-parametric Friedman tests on the coefficient of variance showed significant differences in the perceptual task (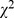
(3) = 24, *p* = 2.2×10^−5^) and in the control task (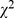
(3) = 15, *p* = 0.0021). The 4 Wilcoxon signed rank tests on the coefficient of variance showed significant influences of stimulus type in both of the discrimination conditions (DR-DS, *W*(11) = 0, *p* = 0.0039) and the identification conditions (IR-IS, *W*(11) = 2, *p* = 0.012). These results show that the variance of the exerted force in the perceptual task is larger for the smooth stimuli than for the rough stimuli. There was no significant difference in the coefficient of variance in the control task found with the 4 Wilcoxon signed rank tests.

#### Scanning velocity

**Figure 8:**
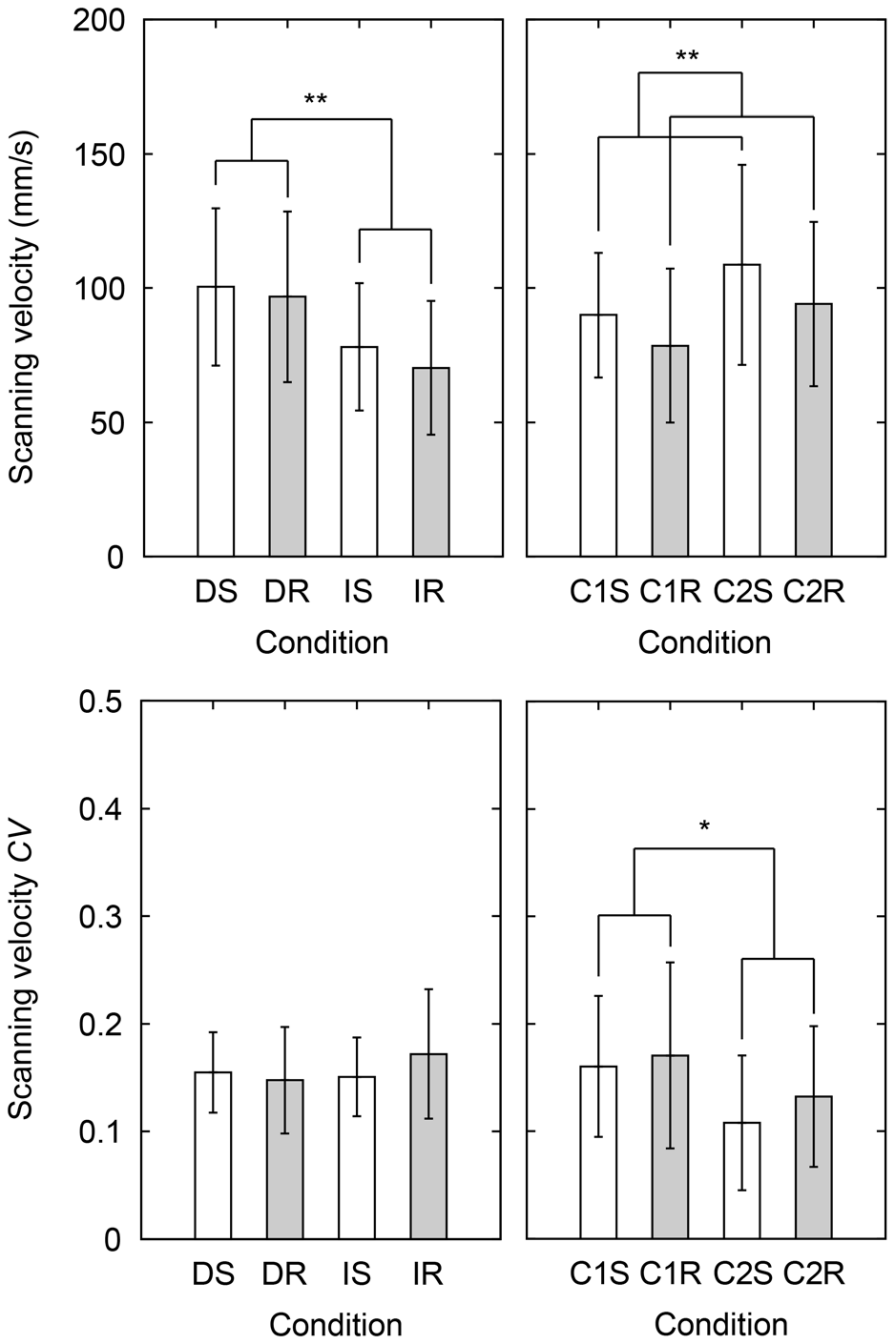
Averages and coefficients of variation of scanning velocity in the various conditions. Left panels: DS, DR, IS, and IR and right panels: C1S, C1R, C2S, and C2R indicate each condition. D, I, C1, and C2 mean discrimination task, identification task, 1^st^ control condition, and 2^nd^ control condition, and S and R mean smooth group and rough group. Error bars indicate the standard deviation. *indicates *p*<0.05 and **p<0.01 with repeated measures ANOVAs.

Figure 8 shows the obtained results on the scanning velocity. An ANOVA showed a significant influence of task in the perceptual conditions (*F*(1,10) = 16, *p* = 0.0024). The result shows that the scanning velocity is larger in the discrimination task than in the identification task. In the control conditions, there is a significant influence of stimulus type (*F*(1,10) = 24, *p* = 6.5×10^−4^). This result shows that scanning velocity used in the control task is larger for the smooth stimuli than for the rough stimuli. For the coefficient of variance in the perceptual task, there are no significant effects of stimulus type, task, nor an interaction effect. An ANOVA on the control task showed that there is a significant difference between the 1^st^ and 2^nd^ conditions (*F*(1,10) = 5.4, *p* = 0.043). This result shows that the variance of the scanning velocity is smaller in the 2^nd^ control condition than in the 1^st^ control condition. There were no significant interactions.

#### Break time

**Figure 9:**
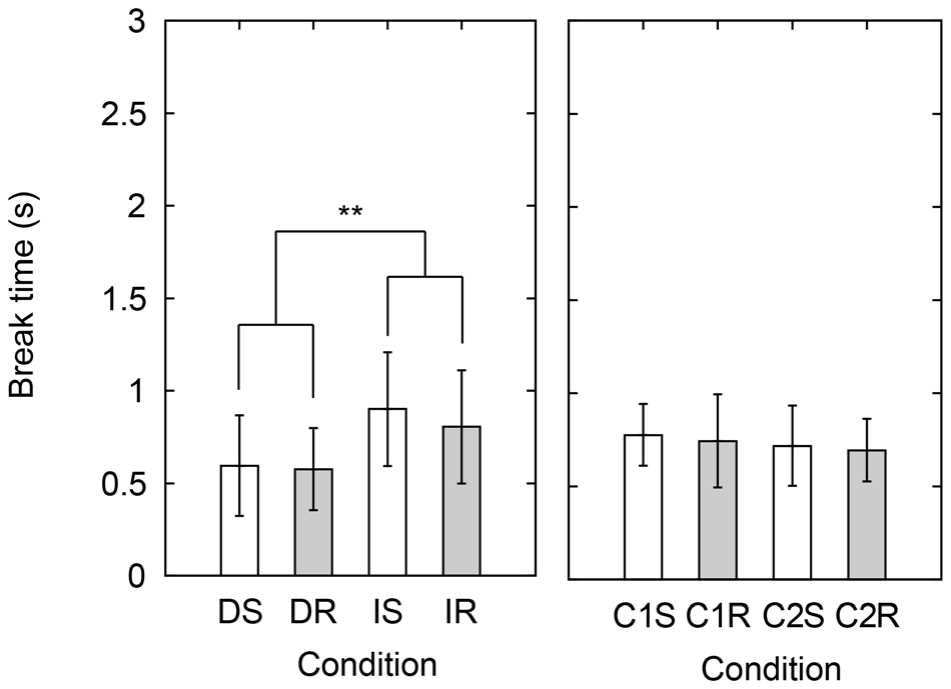
Averages of break time in the various conditions. Averages from all participants except participant 1. Left panels: DS, DR, IS, and IR and right panels: C1S, C1R, C2S, and C2R indicate each condition. D, I, C1, and C2 mean discrimination task, identification task, 1^st^ control condition, and 2^nd^ control condition, and S and R mean smooth group and rough group. Error bars indicate the standard deviation. **indicates *p*<0.01 with repeated measures ANOVAs.

As can be seen in Figure 6, the break time of participant 1 in the identification task seems to be an extreme value. Grubb's test showed that the highest value of 4.9 s (participant 1: IS) is an outlier (*p*<2.2×10^−16^). For this reason, participant 1 was excluded from the analysis of the break time. The result is shown in Figure 9. An ANOVA on the break time showed a significant influence of task in the perceptual conditions (*F*(1,9) = 12, *p* = 0.0065). This result shows that the break time in the discrimination task is shorter than in the identification task.

### Number of Strokes and Difficulty in the Perceptual Tasks

The average of the number of strokes for each condition and its standard deviation were calculated for all participants. The average number of strokes were 10±6 for the discrimination condition with the smooth stimuli, 8±3 for the discrimination condition with the rough stimuli, 6±3 for the identification condition with the smooth stimuli, and 4±1 for the identification condition with the rough stimuli. The average number of strokes for the discrimination task are about twice those for the identification task, because the participants had to touch two stimuli in the discrimination task.

The percentages of correct answers for the rough group and the smooth group were compared in the discrimination task. The average of the correct answers was 88±7% for the rough group and 60±27% for the smooth group. The average of the correct answers for the smooth group showed a large variation, including the percentages of 27.5%, 5%, and 37.5% for participants 1, 5, and 6, respectively. Their correct answers were below the chance level of 50%. It was observed that they tended to discriminate stimuli in the smooth group in the opposite direction: the smooth stimuli S1 and S2 were perceived as rougher than the reference stimulus S3 and the rough stimuli S4 and S5 were perceived as smoother than S3. This was the cause of the low percentages of correct answers. Thus, they might systematically evaluate the roughness of the stimuli in the opposite direction. Very smooth surfaces can be sticky in contact with humans' finger pads. A stick-slip phenomenon generated in the finger pad when it was slid over smooth stimuli might cause the opposite direction judgment. In addition, there might be individual differences in roughness estimation. Hollins, Bensmaïa, Karlof, and Young investigated individual difference in perceptual space for tactile textures. They found two strong dimensions of rough/smooth and hard/soft for all participants, but a third more dimension of sticky/slippery for some participants [30]. Because we wanted to assess discrimination performance, but are less concerned with the actual direction of judgment, the correct answer percentages in the smooth group were converted for participants 1, 5, and 6 to the opposite direction by calculating 1-(the actual correct answer percentage). The revised mean percentage of correct answers in the smooth group was 75±14%. A Shapiro-Wilk test on the data showed that the assumption of a normal distribution was violated. Then, a Wilcoxon signed rank test was conducted on the percentages of correct answers for the rough group and the smooth group. The two sets of scores differed significantly with *W*(11) = 2, *p* = 0.0078. The percentage of correct answers for the rough group was higher than for the smooth group. It follows that the difficulty in the judgment for the roughness of the smooth group was greater than of the rough group.

Similarly, the difficulties in the identification task were assessed: the average percentage correct was 39±13% for the smooth group, and 40±12% for the rough group. For both parameters, the data satisfied the assumption of a normal distribution and homogeneity of variance. A paired *t*-test showed no significant difference between these groups (*t*(10) = 0.22, *p* = 0.83), indicating a comparable difficulty in the identification task. However, it was observed that answer counts were spread out over a larger range in the smooth group than in the rough group, as can be seen in Table 2.

**Table 2:** Average of the answer counts in percent ± standard deviation for each stimulus in the identification task.

		Answer				
Presented stimulus		1	2	3	4	5
Smooth group	S1	40±27	33±21	13±16	5±13	9±14
	S2	26±16	35±16	16±12	20±18	4±8
	S3	13±16	20±18	36±23	15±18	16±20
	S4	5±9	15±16	20±18	36±15	24±15
	S5	4±8	7±13	13±16	31±21	45±34
Rough group	R1	62±19	27±16	9±14	2±6	0
	R2	18±17	33±21	35±20	11±24	4±8
	R3	11±16	35±18	40±20	7±10	7±13
	R4	0	5±13	16±15	33±18	45±16
	R5	0	2±6	15±16	51±23	33±21

### Relation between Roughness and Shear Force

For each stimulus, the average of the shear force was calculated for each participant and for each condition. Next, for each condition and each participant the slope of the linear relation between the average shear force and the log-transformed grain size of the stimuli was estimated using the least-squares method. When data satisfied the assumption of a normal distribution, a *t*-test on the slopes for each condition as compared to zero was conducted using the slopes obtained from all participants. Otherwise, a Wilcoxon signed rank test was conducted. The results show significant slopes between roughness and shear force in the identification condition with the rough stimuli (positive slope, *t*(10) = 3.3, *p* = 0.0080) and in the 2^nd^ control condition with the smooth stimuli (negative slope, *t*(10) = −3.6, *p* = 0.0049).

**Figure 10:**
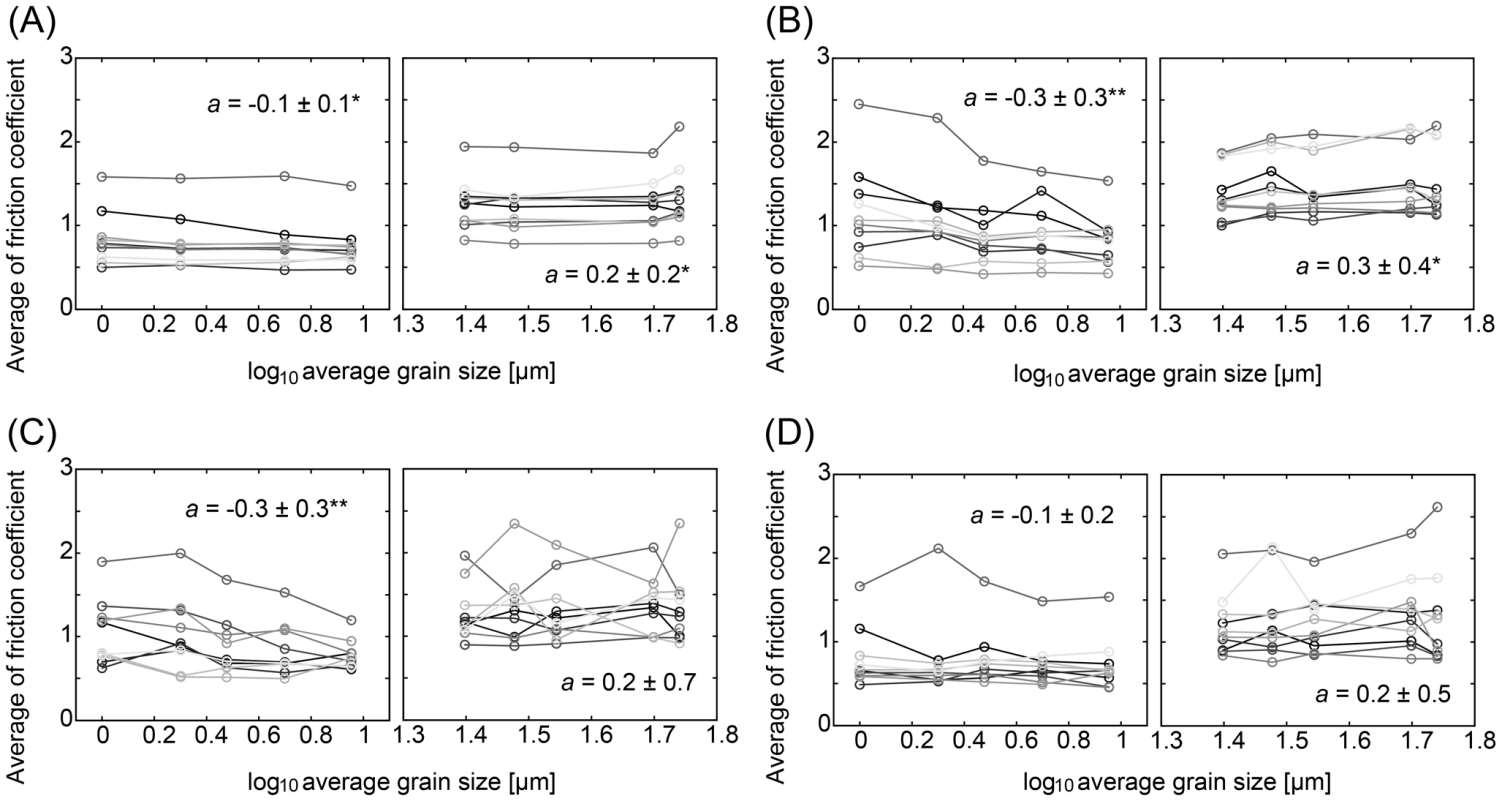
Relationship between friction coefficient and grain size of stimulus. The average of friction coefficients for each participant is shown. (A) Discrimination task. (B) Identification task. (C) 1^st^ control task. (D) 2^nd^ control task. Left panels indicate the results for the smooth stimuli and right panels for the rough stimuli. *a* is the average of the slopes and its standard deviation which were estimated for each participant. *indicates *p*<0.05 and **p<0.01 with *t*-tests or Wilcoxon signed rank tests.

In addition to the shear force, the friction coefficient was also investigated in the same way. A *t*-test on the slopes for each condition as compared to zero or a Wilcoxon signed rank test was conducted. The results show significant slopes between roughness and friction coefficient in all the perceptual conditions: the identification conditions with the rough stimuli (positive slope, *t*(10) = 2.6, *p* = 0.028) and the smooth stimuli (negative slope, *t*(10) = −3.3, *p* = 0.0085); the discrimination conditions with the rough stimuli (positive slope, *t*(10) = 2.6, *p* = 0.025) and the smooth stimuli (negative slope, *W*(11) = 6, *p* = 0.014), and in the 1^st^ control conditions with the smooth stimuli (negative slope, *W*(11) = 0, *p* = 9.8×10^−4^). Figure 10 shows the average of the friction coefficient for each participant. The average of the slopes and its standard deviation for each condition are presented in Figure 10 and significant *p*-values obtained with the *t*-test or the Wilcoxon signed rank test are indicated using asterisks.

## Discussion

First, we will discuss the relation between roughness and shear force or friction coefficient. The normal force exerted by the participants varied substantially in this experiment. As the shear force depends on the normal force, it varied with the exerted normal force. Thus, the shear force did not correlate with grain size of the stimuli in many of the conditions. On the other hand, friction coefficients did correlate with the grain size of the stimuli in all the perceptual conditions. The obtained results support the relationship between the perceived roughness and friction [13], [27]. In particular, slopes were positive for the rough stimuli but negative for the smooth stimuli. A possible reason for the negative slopes is that the surface of the smooth stimuli gets sticky with smaller grain sizes. This result supports the possibility that humans judge the smooth stimuli in the opposite direction, as seen for some participants in the discrimination task. In contrast to the perceptual tasks, for the control task a correlation between grain size and friction coefficient was found only for the smooth stimuli of the 1^st^ control condition. In the control task, the smooth stimuli and the rough stimuli were used interspersed. Thus, the range of the intensity of the stimuli was wider in the control conditions than in the perceptual conditions. Furthermore, the participants were not required to make any judgment in the control task. The participants might want to use a specific movement like keeping the angle of the finger constant for the roughness judgment in the perceptual task, while they did not have to maintain a specific movement in the control task. Consequently, exploratory movements used might have more variance in the control conditions than in the perceptual conditions. The friction coefficient depends not only on the normal force and the shear force but also on the contact area, angle of the finger, and other parameters of the behavior since human fingers have a complex structure and nonlinear physical characteristics. Therefore, correlations with friction coefficients were more likely to be observed in the perceptual conditions.

The three tasks (discrimination, identification, and control) each have a distinct set of neurocognitive requirements (i.e., the amount of mental effort required to perform the task). For discrimination of small differences, perceptual accuracy is important, and the difference between each pair of stimuli is evaluated by exploring two stimuli shortly after one another. In contrast, for the identification task, the classification of the different levels of perceived intensity has to be stored and recalled over a longer period. Lastly, the control task does not involve perception, but only some degree of motor control. The results on the friction coefficient support the difference between the perceptual tasks and the control task. This all will have implications on forces and velocities used. In the following, we will discuss the results and implications for the various parameters.

### Normal Force

The result for the control task showed that the average normal force for the rough stimuli is smaller than that for the smooth stimuli in the 2^nd^ control condition. A possible explanation is aversiveness. The participants were not required to give a response in the control task. Therefore, the obtained results in the control task might have a strong relation with aversiveness. Abrasiveness of coarse sandpaper has a potential to damage the stratum corneum. Alternatively, it is possible that the intensity of the stimulation by the rough stimuli is stronger than that of textures we touch in daily life. Participants might try to achieve some minimum level of sensory input and thus do not require the force to be as high when the input signal is stronger. Considering aversiveness associated with the above points, we might not want to use too large a force for the rough stimuli due to the greater intensity of stimulation. Another explanation is that people tend to keep the same level of stimulation. Large forces might be preferred for the smooth stimuli for the same level of perceived intensity of stimulation as compared to the rough stimuli. The significant difference in the 2^nd^ control condition might be caused by the memory of the presented stimuli and the procedure. Since the 2^nd^ control condition was always conducted in the last session, the participants might predict stimuli and tend to maintain a similar exploratory motion for each stimulus.

From the results on the coefficient of variance, it seems that humans use more variation in normal forces for the smooth stimuli than for the rough stimuli. A possible explanation is a difference in task difficulty due to the stimuli used. The perceived roughness magnitude depends non-linearly on physical roughness. Stevens and Harris [12] found a power law with an exponent of 1.5 for the relation between physical and perceived roughness. In addition, the perceived roughness increases with contact force [16] as mentioned above. Participants might try to supply some level of force for the judgment and change the exerted forces according to the difficulty of each trial. The comparison of correct answers in the discrimination task showed that roughness discrimination for the smooth stimuli was more difficult than for the rough stimuli and the spread of the answers in the identification task indicated that it was more difficult to identify a stimulus among the 5 stimuli for the smooth group than for the rough group. Therefore, greater variation in force is necessary for perceptual tasks in the difficult case (smooth stimuli) for the same level of discriminability as compared to the easy case (rough stimuli). For this reason, a larger variation in force might be expected in the case of smooth stimuli in this experiment. However, the present experiment cannot separate the difficulty from the intensity of stimuli. Considering the intensity of stimuli, the other possible explanation is aversiveness. Humans may use a larger range of forces for the smooth stimuli than for the rough stimuli, before the stimulation becomes unpleasant or even painful. However, the result for the control task showed no significant difference in the variance due to the type of stimulus. Thus, the difference in the variation found for the perceptual tasks might involve the difference in difficulty in addition to some other aspect of the difference between rough and smooth stimuli. In future work, this effect could be investigated using smooth and rough stimuli with equivalent task difficulty.

### Scanning Velocity and Break Time

In the current study, fine-textured surfaces with small grain sizes were used. Recent psychophysical experiments on roughness perception demonstrated that estimation of fine-textured surfaces is based on vibration [18], [19], [20] and in human brain research, it was found that roughness perception differs depending on the scanning velocity [21]. Therefore, the scanning velocity seems to be an important factor for roughness perception as well as the normal force.

From the results of scanning velocity in the control task, it follows that humans use a lower scanning velocity for the rough stimuli than for the smooth stimuli in just exploring without any perceptual objective. A possible explanation is aversiveness as mentioned in the discussion on the normal force. Considering aversiveness, we might not want to use too large a velocity for the rough stimuli due to the greater intensity of stimulation since the perceived roughness increases with scanning velocity [24].

The results on the coefficient of variance showed that the variance of the scanning velocity was smaller in the 2^nd^ control condition than in the 1^st^ control condition. A possible reason is that the participants predict stimuli and tend to maintain a similar exploratory motion.

Furthermore, in Figure 8 it can be seen that within the discrimination task or the identification task, scanning velocity and its coefficient of variance do not differ for the different stimuli. As shown in the previous section, the normal force tends to be changed according to the intensity of the stimuli. As a consequence, it might be a good strategy to not also change the scanning velocity. In contrast to the perceptual tasks, the participants do not have to perceive the roughness in the control task. Therefore, both the normal force and the scanning velocity can be changed according to the intensity of the stimuli. The results on the break time followed the results on the force and the velocity. The result of the break time analysis for the perceptual tasks is consistent with the result on the velocity for the perceptual task in terms of memory requirement. Furthermore, the break time is not significant influenced by stimulus group in the control task. It seems that the break time does not have a relation with the intensity of the stimuli. Kitada et al. demonstrated in an fMRI study that when roughness stimuli were presented to participants, an area of the brain was activated during the estimation task due to the cognitive processing, which was not activated during the no-estimation task [8]. From the results in the current study, differences in exploratory movements between different tasks can be observed. Our results on the normal force, scanning velocity, and break time look consistent and effective for roughness judgment.

In this paper, a relationship between the perceptual objective or the intensity of the texture and the spontaneous touch behavior has been presented. The collected contact normal forces and scanning velocities during active roughness perception showed that exploratory movements seemed to be changed effectively in relation to the perceptual task objective and the task difficulty due to the stimuli used. In future work, experiments on the perceptual performance might make the present discussion stronger. A relationship between the perceptual performance and the spontaneous touch behavior will be investigated by comparison of the performance under spontaneous touch with that under a controlled contact force and scanning velocity different from spontaneous touch, for each participant.
